# Supplementary material for: CellBouncer, a unified toolkit for single-cell demultiplexing and ambient RNA analysis, reveals hominid mitochondrial incompatibilities
Source: Cell Genom. 2026 Jun 10;6(7):101275. doi: 10.1016/j.xgen.2026.101275 (PMC13347948; doi:10.1016/j.xgen.2026.101275)
Supplement: Document S1. Figures S1–S18 and Tables S1–S8 [file mmc1.pdf]

Cell Genomics, Volume *6*

Supplemental information

CellBouncer, a unified toolkit for single-cell demultiplexing and ambient RNA analysis, reveals hominid mitochondrial incompatibilities

Nathan K. Schaefer, Bryan J. Pavlovic, Matthew T. Schmitz, and Alex A. Pollen

Supplementary Material for:

CellBouncer, A Unified Toolkit for Single-Cell Demultiplexing and Ambient RNA Analysis, Reveals Hominid Mitochondrial Incompatibilities

Nathan K. Schaefer[1,2,3,5]*, Bryan J. Pavlovic[1,2,3]*, Matthew T. Schmitz [4], and Alex A. Pollen[1,2,3]

[1]The Eli and Edythe Broad Center of Regeneration Medicine and Stem Cell Research, University of California San Francisco, San Francisco, CA 94143, USA

[2]Department of Neurology, University of California San Francisco, San Francisco, CA 94143, USA

[3]Weill Institute for Neurosciences, University of California San Francisco, San Francisco, CA 94158, USA

[4]Allen Institute for Brain Science, Seattle, WA 98109, USA

[5]Lead Contact

* These authors contributed equally.

*Correspondence: nkschaef@gmail.com (N.K.S), alex.pollen@ucsf.edu (A.A.P.)

Supplementary figures

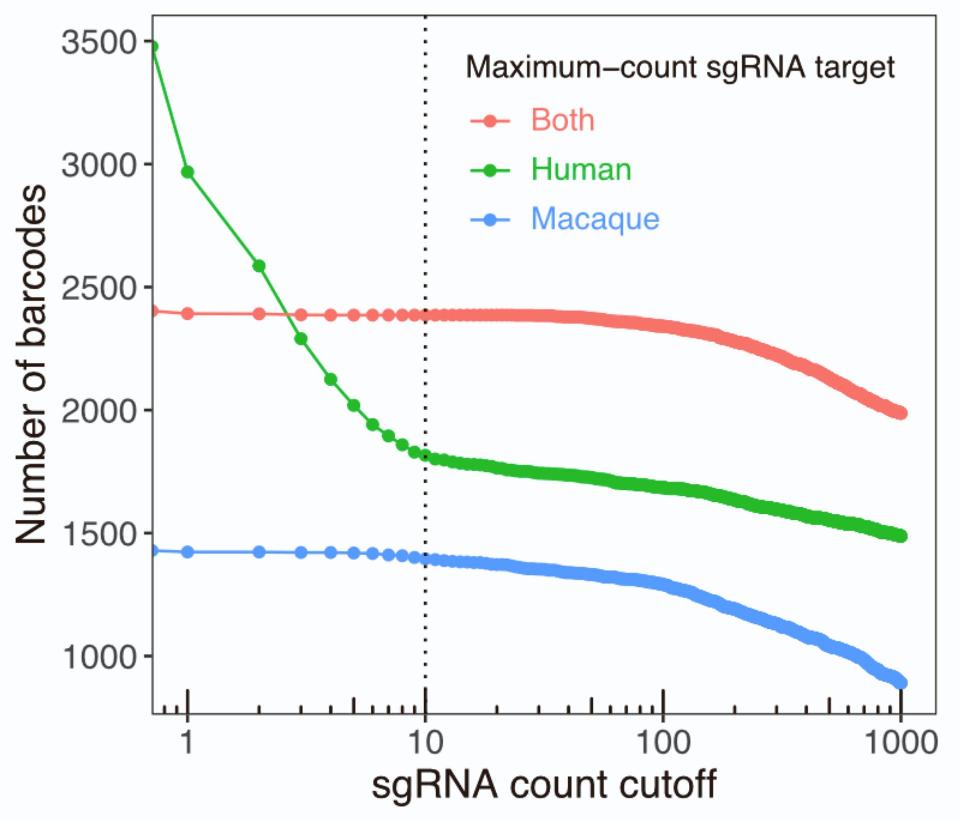

Figure S1. Choosing sgRNA count thresholds for ground truth species assignments in pooled species dataset, related to STAR Methods

Determining a minimum sgRNA count for reliable estimation of ground truth species identity per cell in the pooled species dataset[1]. Points are cell barcodes colored by the species target of the highest-count sgRNA associated with the cell (sgRNAs can target human-specific, cynomolgus macaque-specific, or non-specific sequences). X-axis: sgRNA count cutoff; Y-axis: number of cell barcodes with the maximum-count sgRNA belonging to the shown genome. Barcode counts stabilize above the chosen cutoff of 10 sgRNA counts per cell.

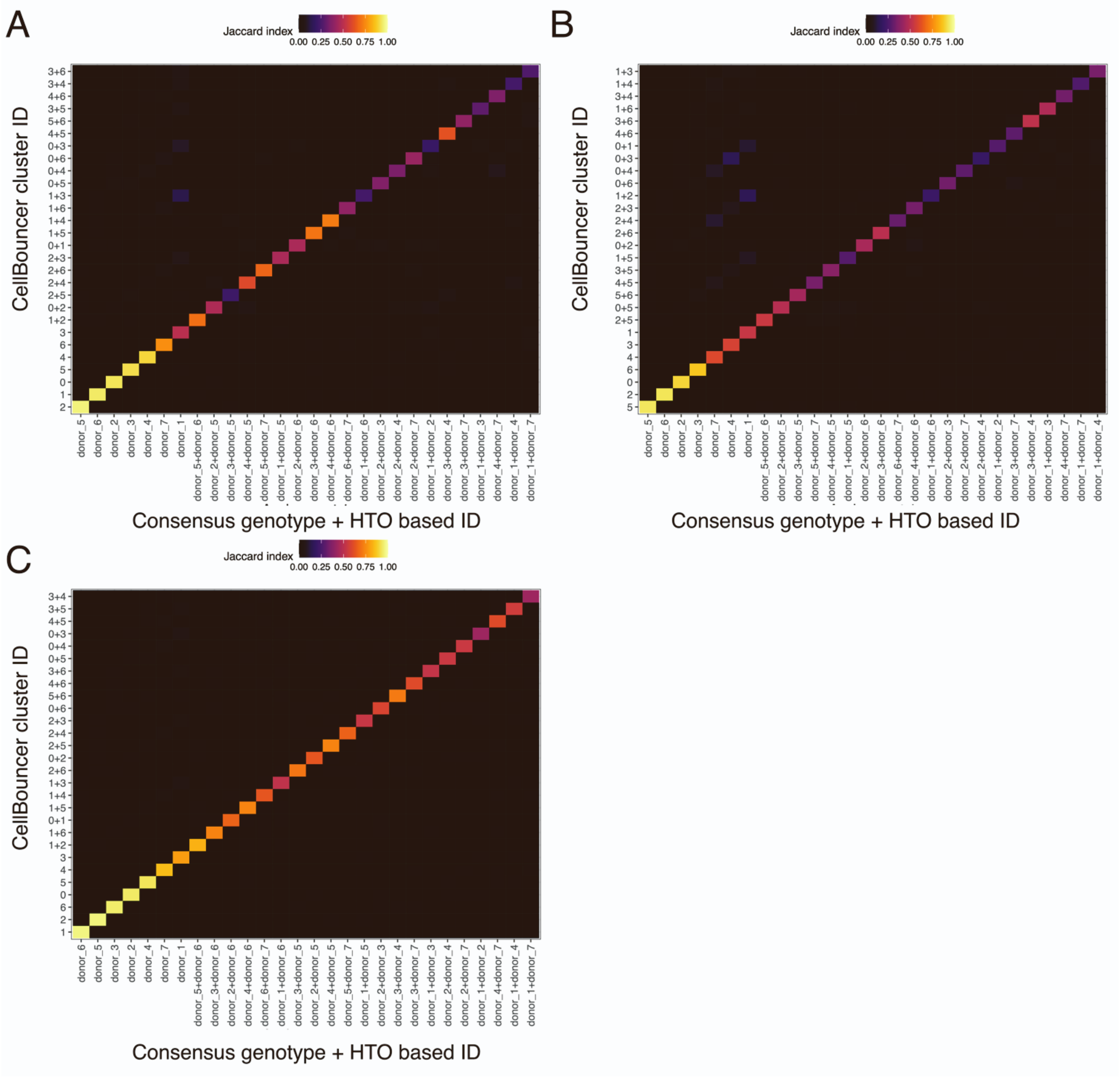

Figure S2. Accuracy of clustering-based cell identities produced by CellBouncer's MT-to-VCF pipeline, related to Figure 3

Comparison of cell labels from consensus genotype and CellPlex-based assignments (X-axis) vs inferred assignments without prior genotype data, using CellBouncer's MT-to-VCF pipeline. Color denotes Jaccard similarity between the group of cells with the label on the X and the Y axis; doublet identities are named as component singlet identities separated by "+". A: 2,000 cell downsampled dataset. B: 5,000 cell downsampled dataset. C: full dataset (~33,000 cells).

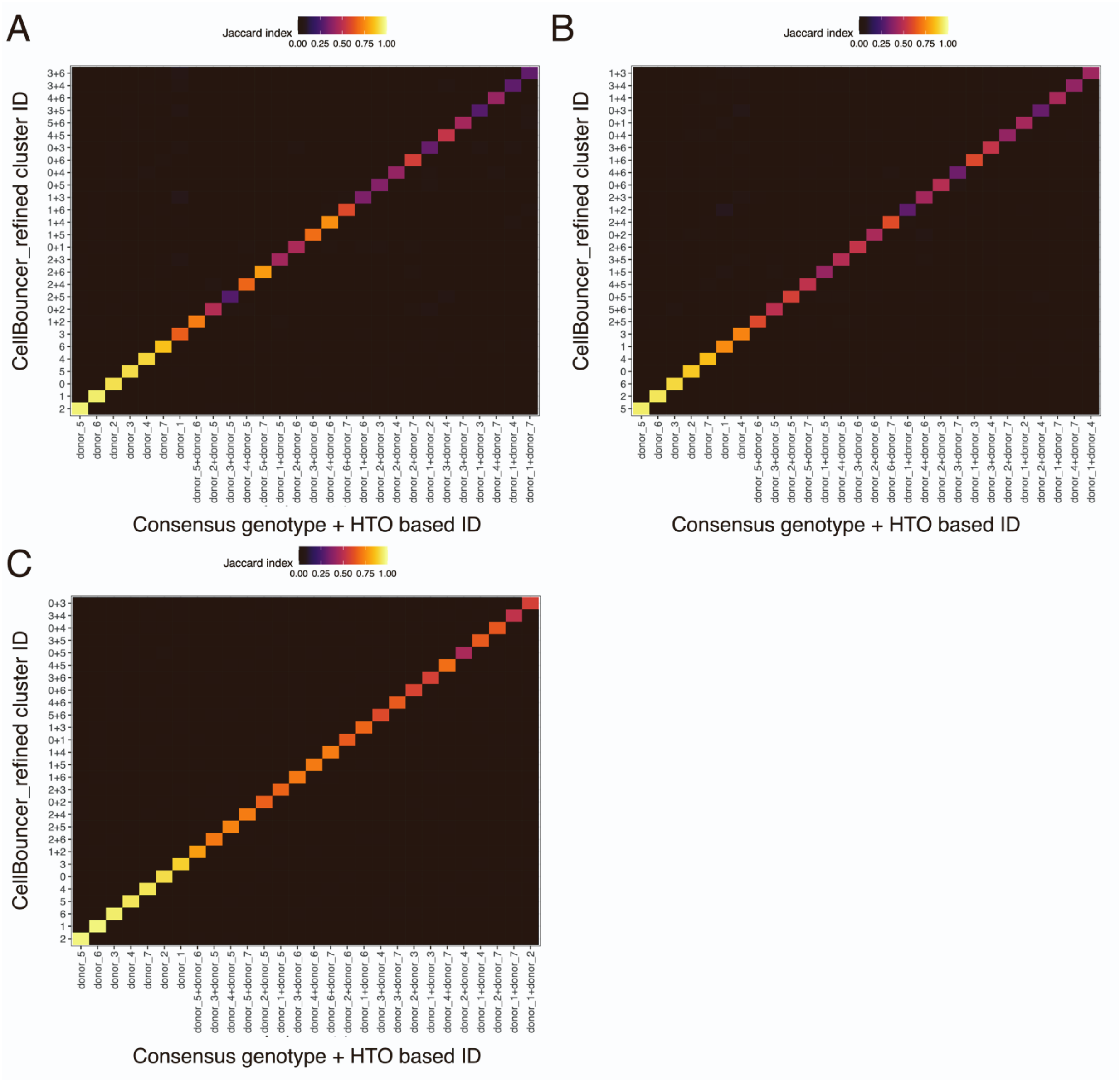

Figure S3. Accuracy of clustering-based cell identities produced by CellBouncer's MT-to-VCF pipeline with genotype refinement, related to Figure 3

Comparison of cell labels from consensus genotype and CellPlex-based assignments (X-axis) vs inferred assignments without prior genotype data, using CellBouncer's MT-to-VCF pipeline, followed by refine_vcf to re-infer genotypes given assignments, and another run of demux_vcf using the refined genotypes. Color denotes Jaccard similarity between the group of cells with the label on the X and the Y axis; doublet identities are named as component singlet identities separated by "+". A: 2,000 cell downsampled dataset. B: 5,000 cell downsampled dataset. C: full dataset (~33,000 cells).

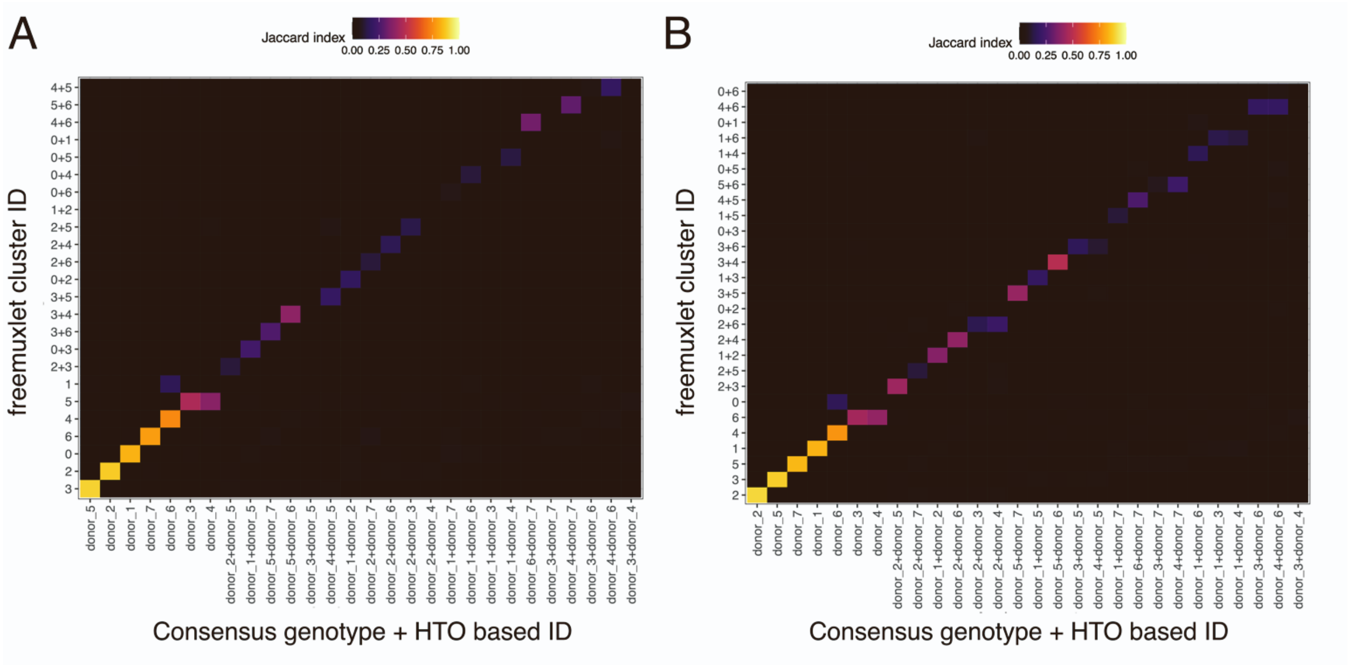

Figure S4. Accuracy of clustering-based cell identities produced by freemuxlet, related to Figure 3

Comparison of cell labels from consensus genotype and CellPlex-based assignments (X-axis) vs inferred assignments without prior genotype data, using freemuxlet[2]. Color denotes Jaccard similarity between the group of cells with the label on the X and the Y axis; doublet identities are named as component singlet identities separated by “+”. A: 2,000 cell downsampled dataset. B: 5,000 cell downsampled dataset. Full dataset is missing because the run did not complete.

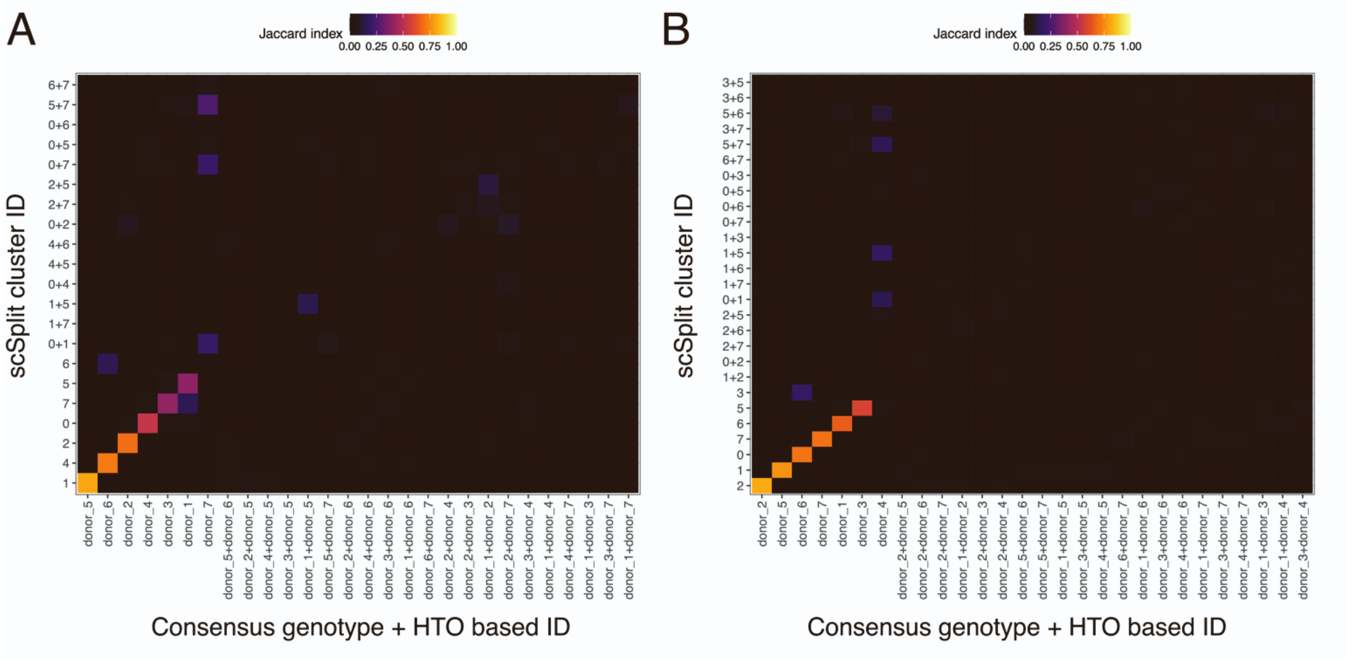

Figure S5. Accuracy of clustering-based cell identities produced by scSplit, related to Figure 3

Comparison of cell labels from consensus genotype and CellPlex-based assignments (X-axis) vs inferred assignments without prior genotype data, using scSplit[3]. Color denotes Jaccard similarity between the group of cells with the label on the X and the Y axis; doublet identities are named as component singlet identities separated by "+". A: 2,000 cell downsampled dataset. B: 5,000 cell downsampled dataset. Full dataset is missing because the run did not complete.

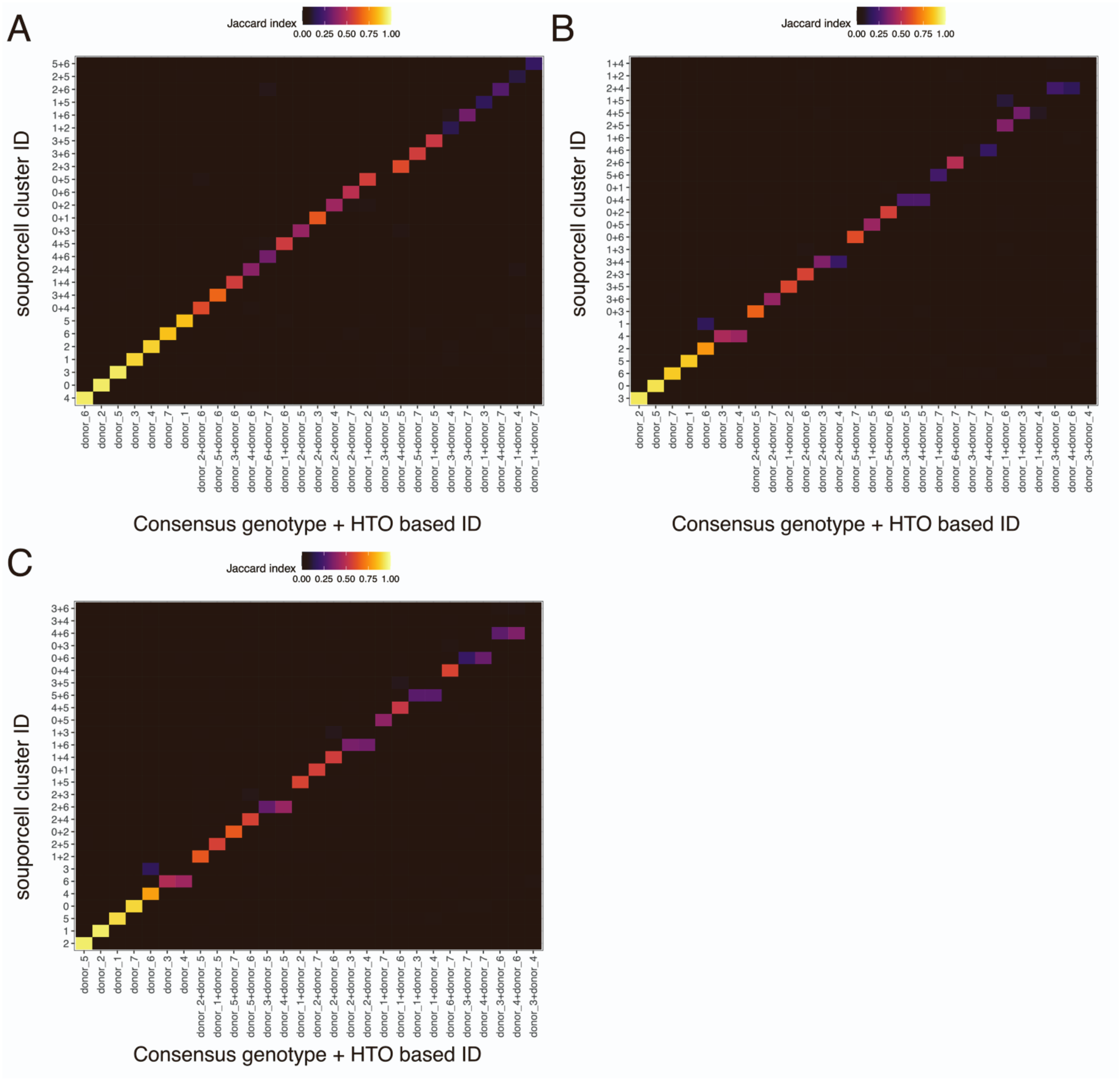

Figure S6. Accuracy of clustering-based cell identities produced by souporcell, related to Figure 3

Comparison of cell labels from consensus genotype and CellPlex-based assignments (X-axis) vs inferred assignments without prior genotype data, using souporcell[4]. Color denotes Jaccard similarity between the group of cells with the label on the X and the Y axis; doublet identities are named as component singlet identities separated by “+”. A: 2,000 cell downsampled dataset. B: 5,000 cell downsampled dataset. C: full dataset (~33,000 cells).

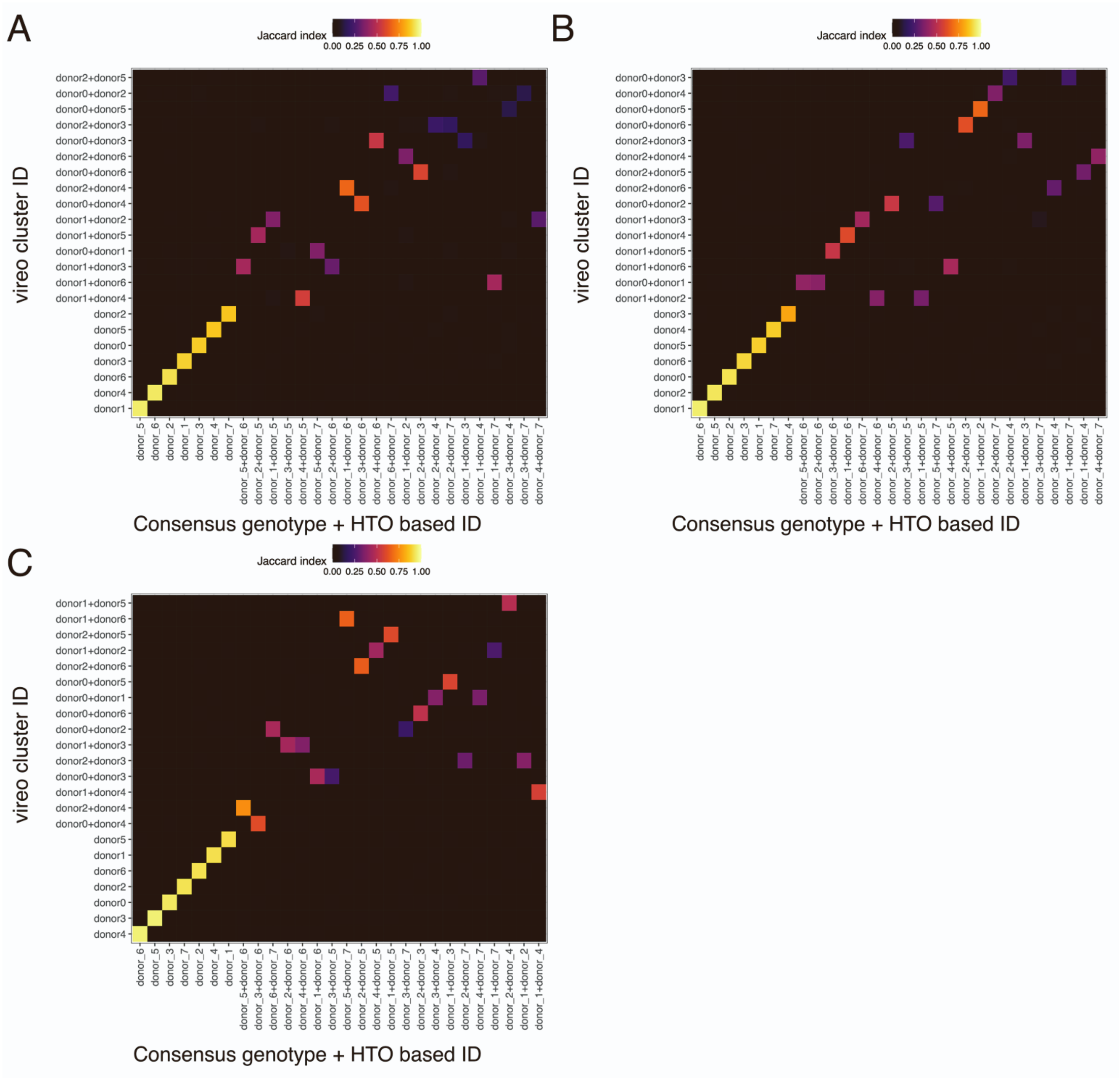

Figure S7. Accuracy of clustering-based cell identities produced by Vireo, related to Figure 3

Comparison of cell labels from consensus genotype and CellPlex-based assignments (X-axis) vs inferred assignments without prior genotype data, using Vireo[5]. Color denotes Jaccard similarity between the group of cells with the label on the X and the Y axis; doublet identities are named as component singlet identities separated by “+”. A: 2,000 cell downsampled dataset. B: 5,000 cell downsampled dataset. C: full dataset (~33,000 cells).

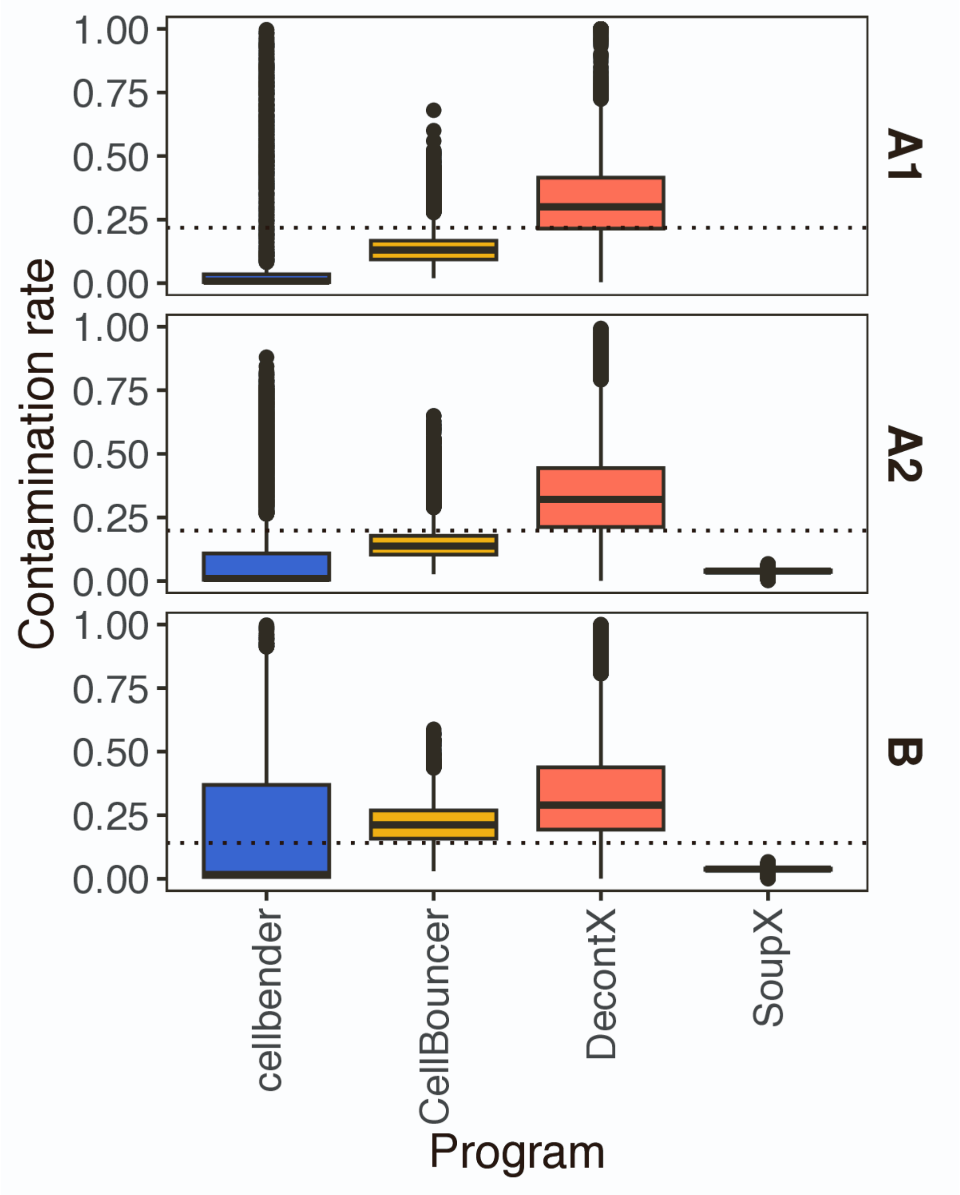

Figure S8. Per-cell ambient RNA contamination rates in composite iPSC scRNA-seq data inferred by four programs, related to STAR Methods

Comparison of inferred per-cell contamination rates using four programs (CellBouncer, cellbender, DecontX, and SoupX), on three single cell RNA-seq libraries from tetraploid composite cell lines. The dotted lines are estimates produced by comparing inferred bulk proportions to the proportions of cells assigned to each identity using demux_vcf (see Methods). Panels are libraries; A1 and A2 are replicates of the same library and B is a single replicate of another library. SoupX was unable to run on library A1.

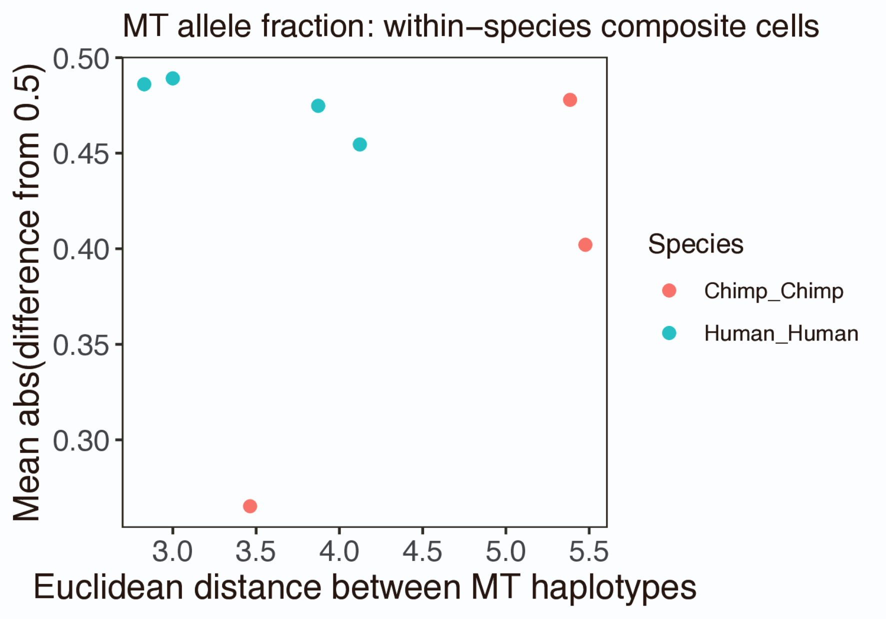

Figure S9. Greater sequence diversity among chimpanzee than human mitochondrial haplotypes does not explain the finding that chimpanzee/chimpanzee composite iPSCs retain mitochondria from both contributors more often than human/human composite iPSCs, related to Figure 6

Relationship between the similarity of mitochondrial haplotypes of contributor lines and ratios of read counts matching each contributor line's mitochondrial haplotype in within-species composite cells. X-axis: Euclidean distance between the two mitochondrial haplotypes in a composite cell line (computed using the dist function in R after encoding each mitochondrial haplotype as a bit string, with the ancestral allele = 0 and derived allele = 1 at each SNP). Y-axis: mean divergence in mitochondrial read fraction from expectation under equal proportions of each mitochondrion (0.5), in composite cells. Read fractions are count1/(count1+count2), where count1 is the number of reads matching contributor line 1's mitochondrial haplotype and count2 is the number of reads matching contributor line 2's mitochondrial haplotype, only considering SNPs that segregate between the two contributor mitochondrial haplotypes. This plot shows that species differences in the number of cells retaining mitochondria from both contributor lines are unlikely due to lower numbers of SNPs segregating between human than chimpanzee mitochondrial haplotypes, and therefore unlikely to be a technical artifact.

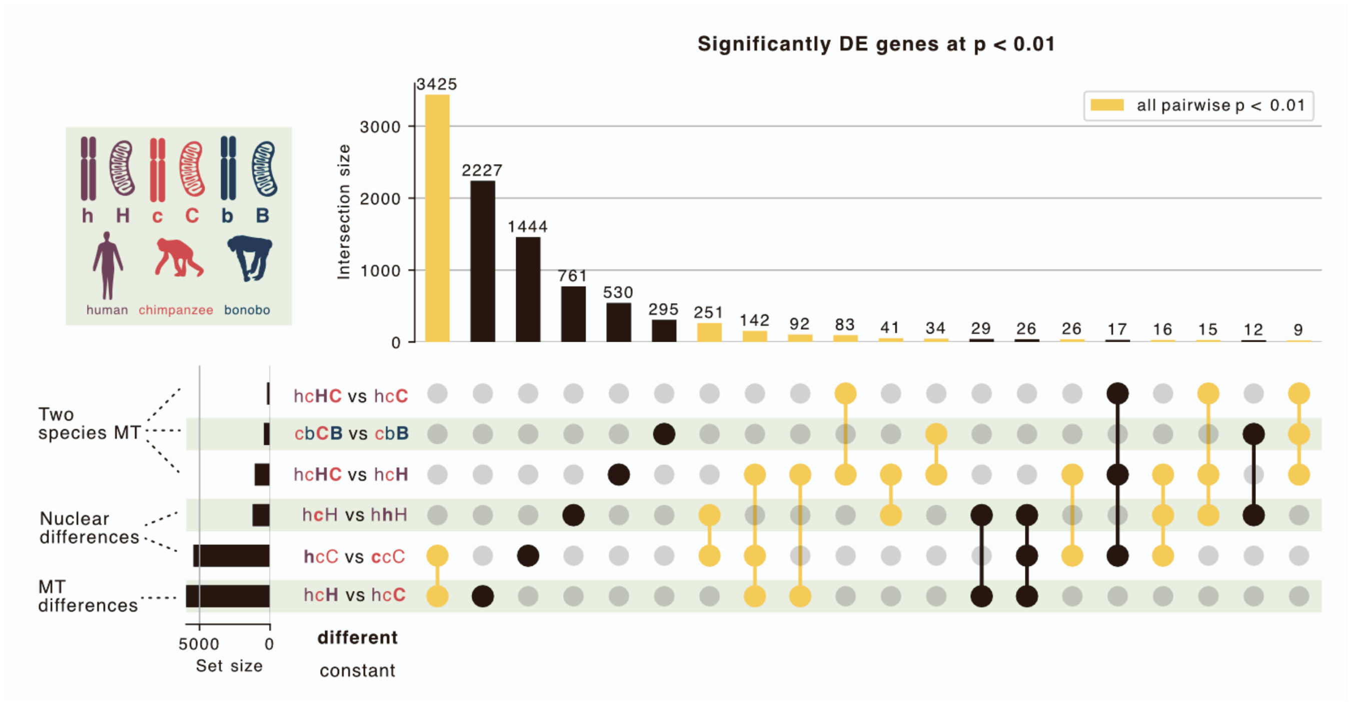

Figure S10. Sizes of differentially expressed gene sets comparing composite iPSCs grouped by nuclear and mitochondrial species of origin, related to Figure 6

UpSet plot[6] showing overlap of sets of differentially expressed genes between different classes of cells, created using the upset_enrich Python package (https://github.com/nkschaefer/upset_enrich). For each comparison, cells with p < 0.01 of differential expression were selected, and the set of significantly DE genes for each cell class was tested for overlap with all others. Sets were highlighted in yellow when all pairwise comparisons between member gene sets were significant (p < 0.01) according to the hypergeometric CDF. Each cell class is named according to the legend; bold text highlights the difference in each comparison. This plot shows significant concordance between responses to having two species' mitochondria and hints that the human genome may mount a larger transcriptional response to the presence of chimpanzee mitochondria than the chimpanzee genome's response to the presence of human mitochondria.

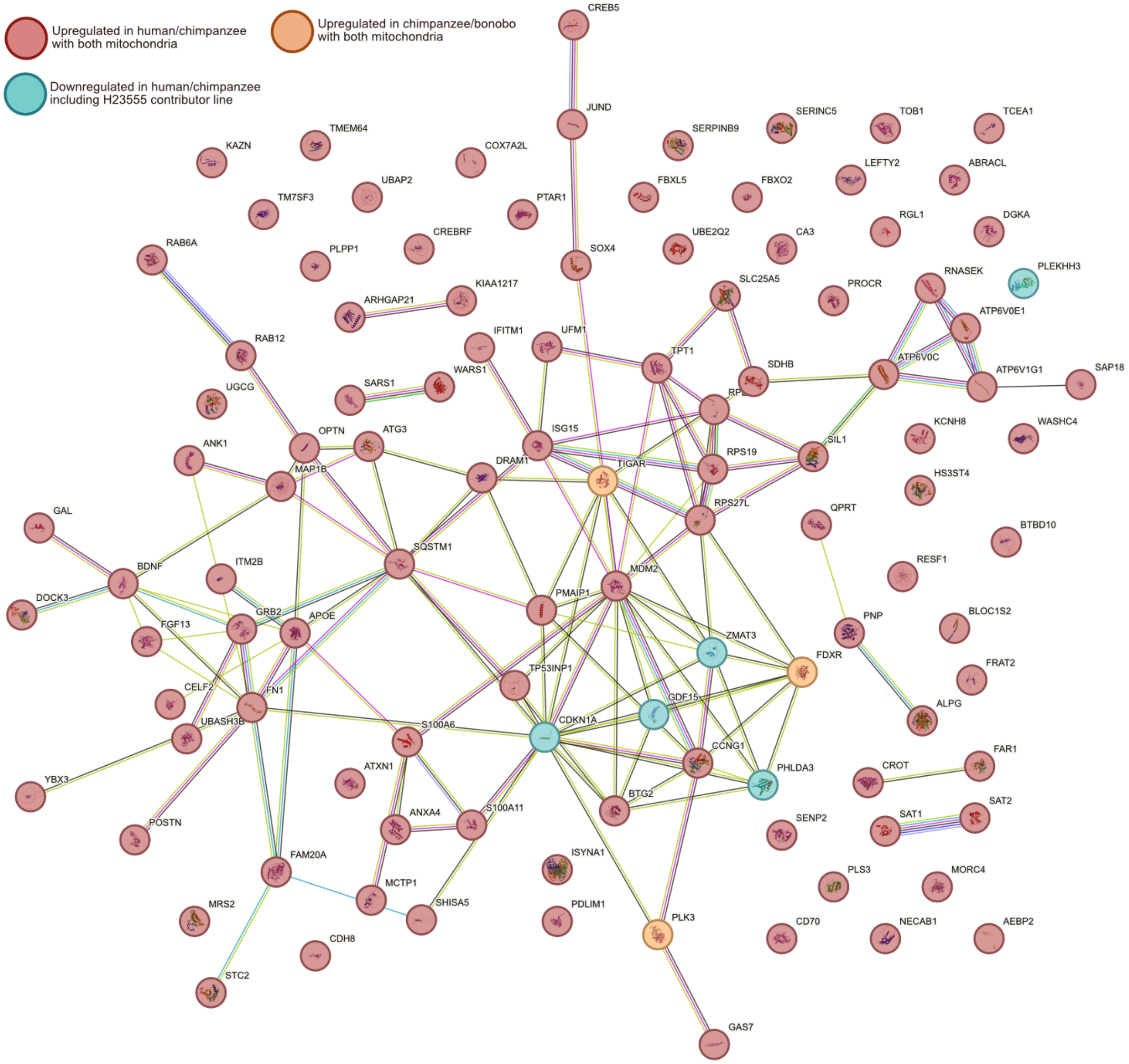

Figure S11. Interaction network for genes significantly differentially expressed in response to the presence of two species' mitochondria within the same cell, related to Figure 6

STRING[7] network showing all genes significantly upregulated in response to having both human and chimpanzee mitochondria ($p < 0.01$, $\log_2$ fold change > 0 comparing human/chimpanzee cells with both species' mitochondria to both human/chimpanzee cells with human mitochondria and human/chimpanzee cells with chimpanzee mitochondria). Protein-protein interactions ($p = 4.11 \times 10^{-11}$), and genes involved in p53 signaling ($p = 0.022$) are enriched in this set. Orange genes are also upregulated ($p < 0.01$) in chimpanzee/bonobo cells with both species' mitochondria relative to those with only bonobo mitochondria (enriched

for overlap with this set, $p = 1.34 \times 10^{-8}$). Teal genes were downregulated ($p < 0.01$) in human/chimpanzee cells from contributor line H23555, relative to other human/chimpanzee cells (enriched for overlap with this set, $p \approx 0$). This plot reveals a key part of the regulatory network involved in p53 signaling that is activated when two species' mitochondria are present, and deactivated by cells capable of surviving with two species' mitochondria.

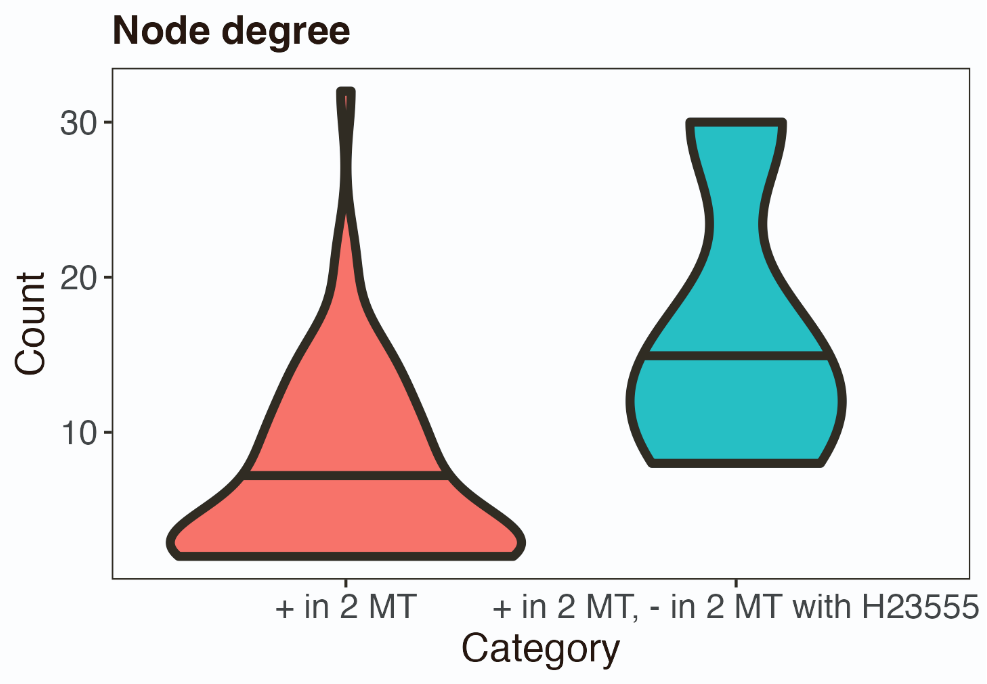

Figure S12. Genes responsive to the presence of two species' mitochondria in the same cell, but downregulated in cells derived from contributor line H23555, show high connectivity in the interaction network, related to Figure 6

Number of edges (degree or connectivity) of nodes in Figure S16, colored by whether or not they were downregulated in cells derived from the contributor line H23555. Genes downregulated in human/chimpanzee cells derived from contributor line H23555, relative to those that did not derive from this line, not only downregulated a significant number of genes upregulated in human/chimpanzee cells with two species' mitochondria, but the downregulated genes tended to be higher-connectivity "hub" genes (Kolmogorov-Smirnov p = 0.05392). This suggests that these genes may be key regulators in the transcriptional response to the presence of two species' mitochondria.

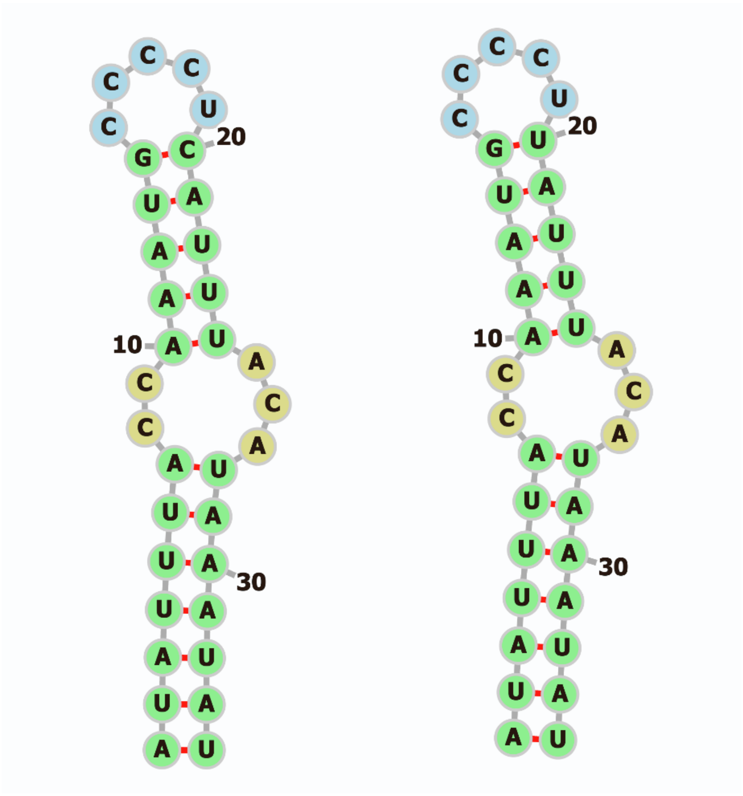

Figure S13. The human-derived T-C mutation at chrM:10479 increases the probability of base pairing within a predicted hairpin structure, related to Figure 6

Predicted hairpin-loop secondary structure from RNAfold[8] for the region around fixed the human-specific derived T-C mutation at chrM:10479 (hg38). This region (chrM:10459-10492 inclusive) was chosen by predicting structure for a broader region and selecting bases involved in the hairpin structure around the focal site. The mutation of interest is labeled as base position 20 in both structures. Left: human reference sequence; Right: human (hg38) reference sequence with the base back-mutated to the ancestral allele. Predicted base pairing probability between base 14 and 20 in the human (left) structure is 0.948, while the same probability with the ancestral allele is 0.611.

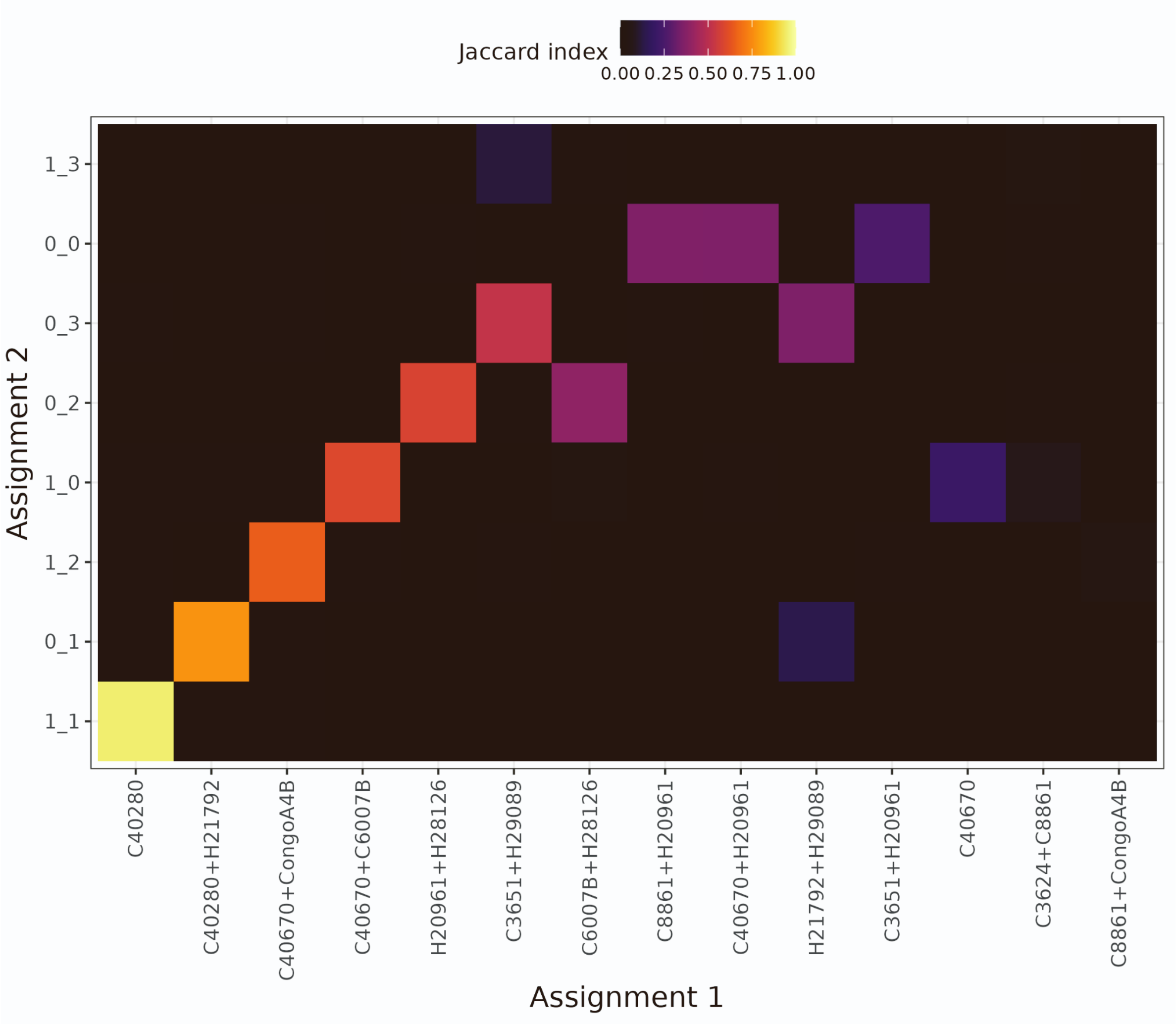

Figure S14. Agreement of genotype-based and mitochondrial haplotype clustering-based identities in composite iPSC library A1, related to STAR Methods

Genotype-based identities from demux_vcf (X-axis) versus inferred mitochondrial haplotypes using demux_mt with one round of subclustering (Y-axis) for tetraploid composite RNA-seq library A1. Subcluster identities are numeric IDs from each round of subclustering, separated by underscores. Colors denote Jaccard index of overlap between the set of cells belonging to the identity on the X and the identity on the Y axis.

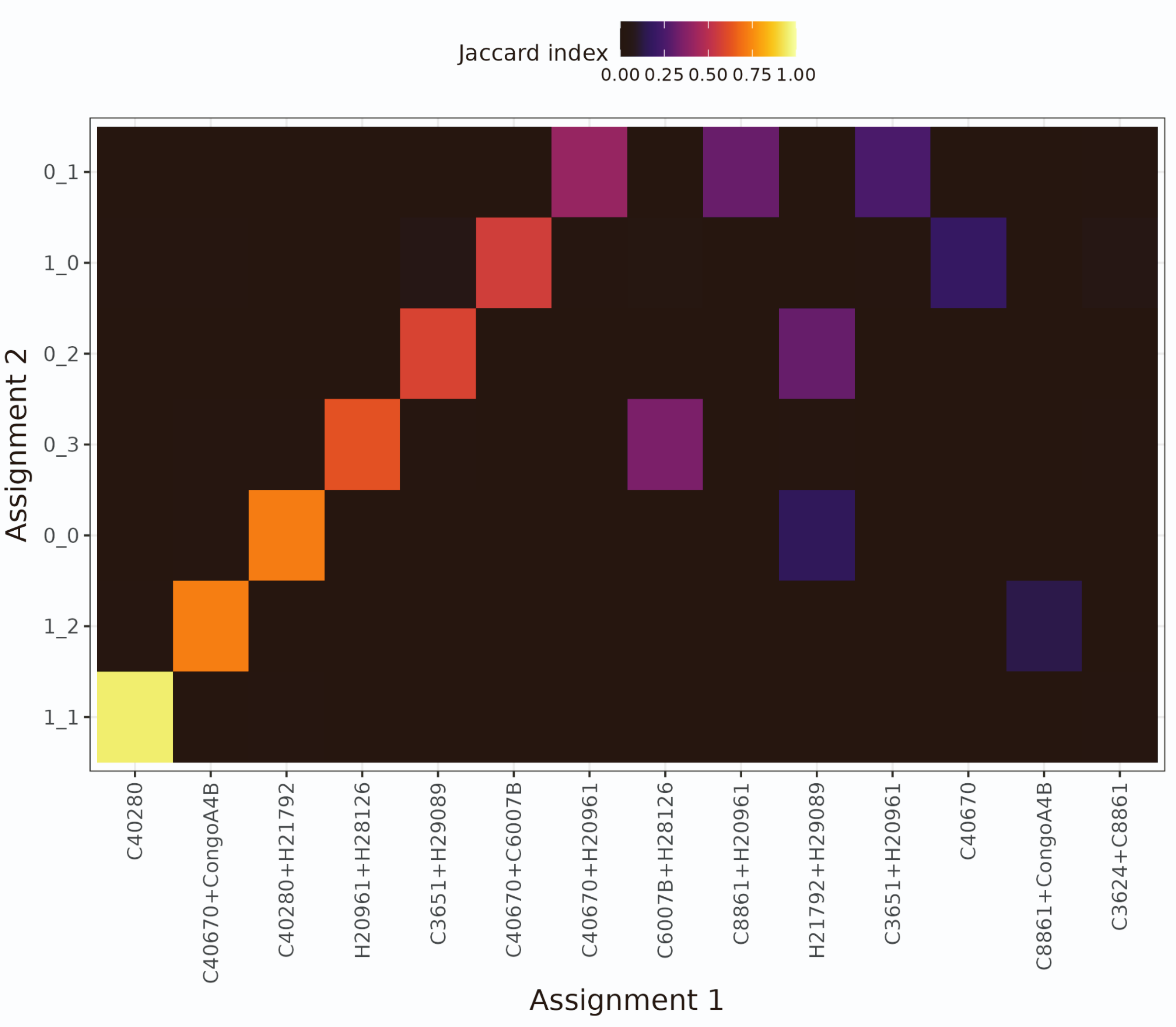

Figure S15. Agreement of genotype-based and mitochondrial haplotype clustering-based identities in composite iPSC library A2, related to STAR Methods

Genotype-based identities from demux_vcf (X-axis) versus inferred mitochondrial haplotypes using demux_mt with one round of subclustering (Y-axis) for tetraploid composite RNA-seq library A2. Subcluster identities are numeric IDs from each round of subclustering, separated by underscores. Colors denote Jaccard index of overlap between the set of cells belonging to the identity on the X and the identity on the Y axis.

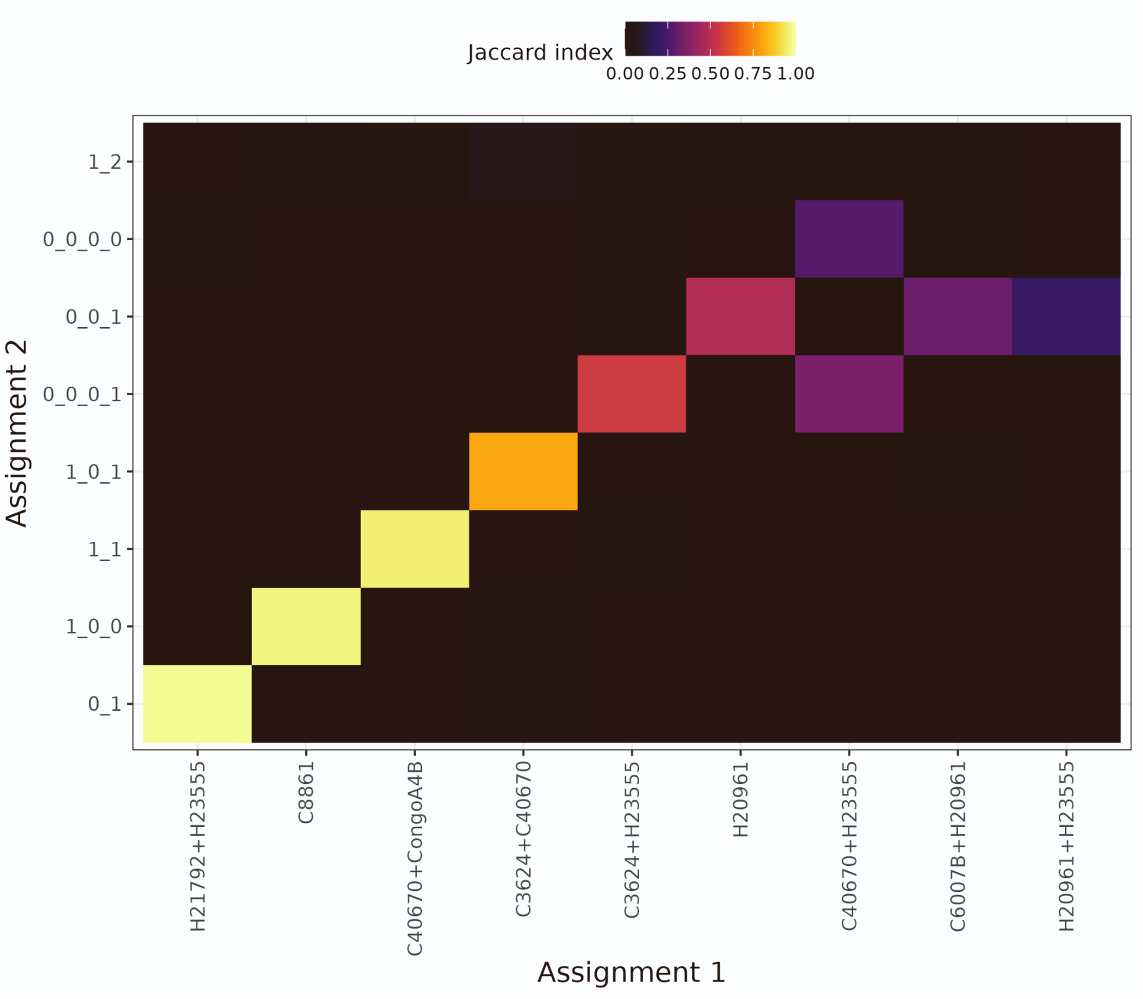

Figure S16. Agreement of genotype-based and mitochondrial haplotype clustering-based identities in composite iPSC library B, related to STAR Methods

Genotype-based identities from demux_vcf (X-axis) versus inferred mitochondrial haplotypes using demux_mt with three rounds of subclustering (Y-axis) for tetraploid composite RNA-seq library B. Subcluster identities are numeric IDs from each round of subclustering, separated by underscores. Colors denote Jaccard index of overlap between the set of cells belonging to the identity on the X and the identity on the Y axis.

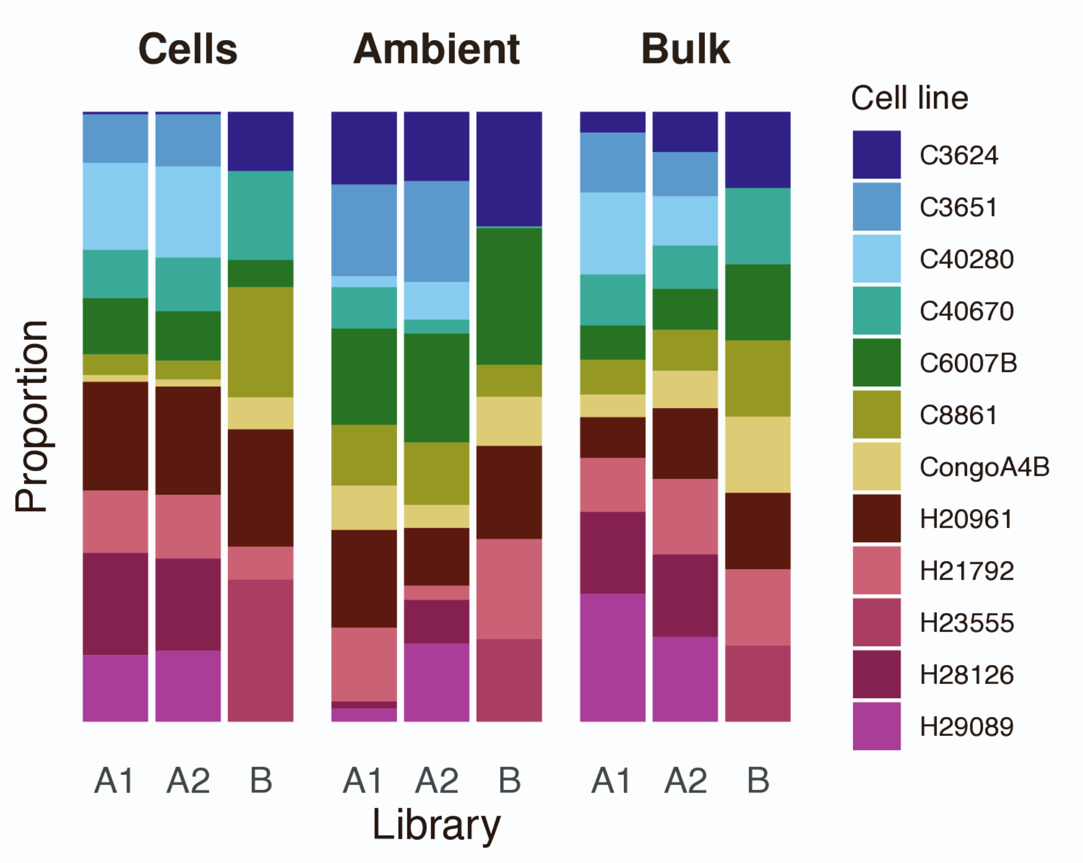

Figure S17. CellBouncer-inferred cell identity, ambient RNA, and overall (bulk) pool proportions in composite iPSC scRNA-seq data, related to STAR Methods

Proportions of total RNA from each contributor cell line from cell assignments using demux_vcf (left panel), proportion of ambient RNA inferred to originate from each contributor cell line (middle panel), and proportion of bulk RNA inferred to come from each contributor cell line (right panel), in three single cell RNA-seq libraries produced from tetraploid composite cell lines. A1 and A2 are technical replicates of the same library, B is a single replicate of a different library, and cell lines beginning “C” are chimpanzee lines (with the exception of CongoA4B, a bonobo cell line), and cell lines beginning “H” are human cell lines.

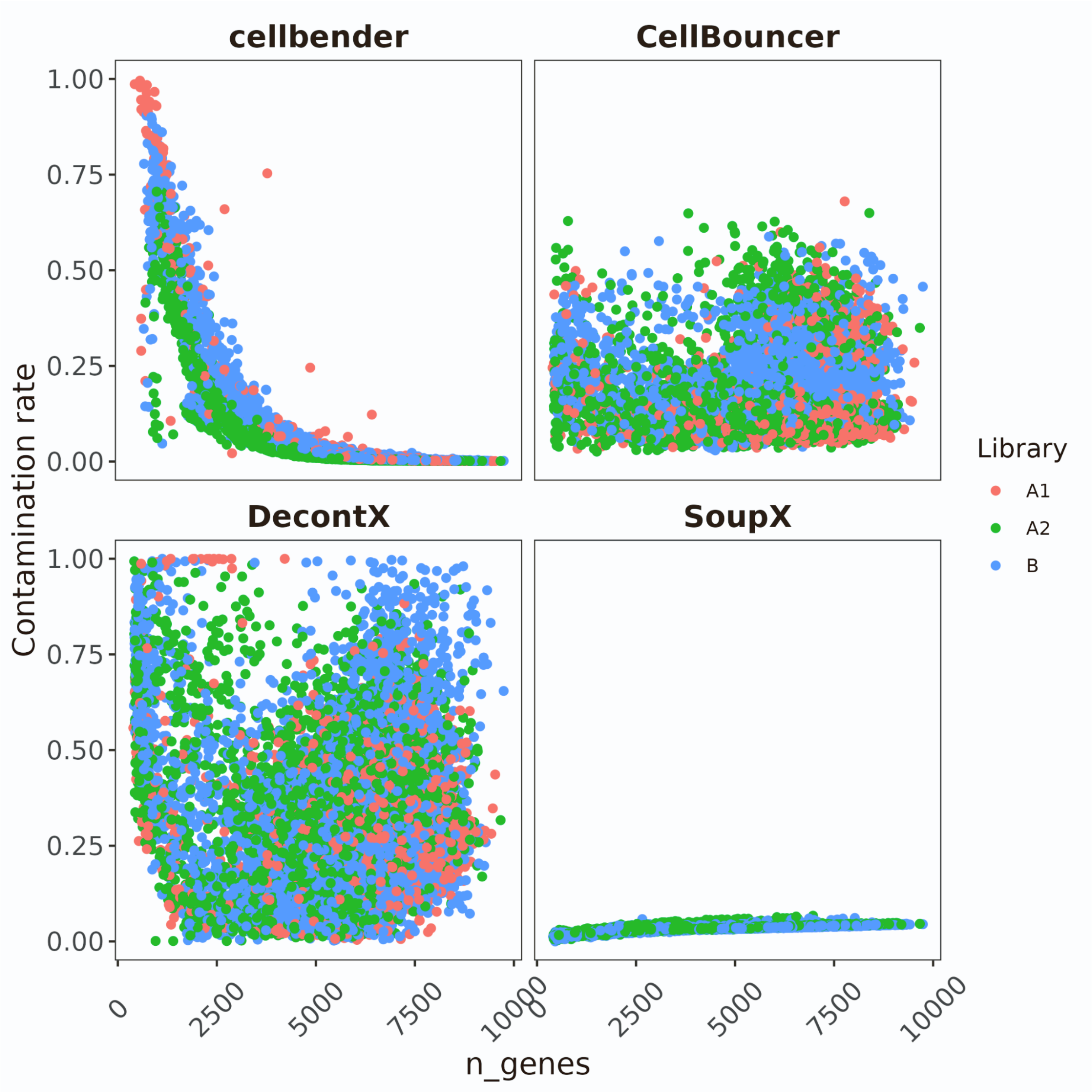

Figure S18. Relationship between inferred per-cell ambient RNA contamination rate and number of genes detected per cell in composite iPSC scRNA-seq data, related to STAR Methods

Comparison of per-cell contamination rate estimates inferred using four programs (cellbender, CellBouncer, DecontX, and SoupX) to the number of genes detected per cell in three single-cell RNA-seq libraries produced from tetraploid composite cell lines. A1 and A2 are technical replicates of the same library and B is a single replicate of a different library. SoupX was unable to run on library A1.

Supplementary Tables

k	k-mers sampled per species	Barcodes assigned	Publication-filtered cells assigned	CellBouncer-filtered cells assigned	Assignments correct	Publication-filtered assignments correct	CellBouncer-filtered assignments correct
15	all	4040055	2888	11343	51.04%	30.23%	18.35 %
20	all	1898904	2888	9799	84.54%	100.00%	99.86%
25	all	1508958	2888	6917	93.67%	99.97%	99.81%
35	all	1204279	2888	7124	96.01%	99.93%	99.83%
45	all	981432	2888	8376	97.26%	99.90%	99.82%
20	1.00E+06	1324085	2888	5423	95.18%	100.00%	99.91%
20	5.00E+06	1674776	2888	8635	91.04%	100.00%	99.86%
20	1.00E+07	1746481	2888	9211	89.13%	100.00%	99.85%
20	2.00E+07	1833455	2888	9637	86.31%	100.00%	99.85%

Table S1. Accuracy of demux_species on a labeled dataset, related to Figure 2

Data are from a published study[9] including cells from three species processed separately (so the true species identities are known). 300 artificial interspecies doublets were also included. Publication-filtered cells refer to cell barcodes that passed quality control after alignment in the published study. CellBouncer-filtered cells refer to cell barcodes that CellBouncer inferred contain real cells based on their species-specific k-mer counts. The “Assignments correct” column includes cell barcodes that do not represent true cells.

Reference panel size (number of SNPs)							
**Program**	**1,000**	**10,000**	**25,000**	**50,000**	**100,000**	**250,000**	**511,405**
CellBouncer	0.138	0.566	0.764	0.837	0.899	0.945	0.969
demuxalot	0.229	0.666	0.835	0.884	0.927	0.953	0.965
demuxlet	0.212	0.574	0.789	0.838	0.898	0.949	0.98
vireo	0.009	0.469	0.743	0.812	0.849	0.872	0.875
**Number of cells**							
**Program**	**100**	**500**	**1,000**	**5,000**	**10,000**	**20,000**	**32,669**
CellBouncer	0.98	0.971	0.967	0.972	0.967	0.969	0.969
demuxalot	0.95	0.962	0.963	0.966	0.964	0.964	0.965
demuxlet	0.96	0.98	0.979	0.974	0.98	0.98	0.98
vireo	0.812	0.865	0.873	0.864	0.871	0.875	0.875
**Sequencing depth (expected reads per cell)**							
**Program**	**5,000**	**12,500**	**25,000**	**37,500**	**50,000**		
CellBouncer	0.884	0.932	0.954	0.962	0.969		
demuxalot	0.921	0.948	0.959	0.962	0.965		
demuxlet	0.922	0.953	0.968	0.974	0.98		
vireo	0.823	0.861	0.871	0.873	0.875		

Table S2. Accuracy of demux_vcf compared to three alternative tools on a benchmarking dataset, related to Figure 3

F-score, or the harmonic mean of precision and recall, of CellBouncer against three alternative tools on 10X Genomics 40,000 cell 7-donor NSCLC single-cell RNA-seq dataset[10]. Ground truth was the majority vote assignment across all methods plus 10X CellPlex data; ties were broken using CellPlex assignments. Top section: effect of randomly downsampling the genotype data to the reported number of SNPs (right column uses the full panel). Middle section: effect of randomly downsampling the number of cells in the dataset (right column uses all cells). Bottom section: effect of randomly downsampling reads to the reported targeted depth per cell (right column uses all reads).

Method	Error	Dataset	Precision	Recall	F score	Num. variants
CellBouncer		Full	0.925	0.923	0.924	379,319
CellBouncer + refine_vcf		Full	0.939	0.937	0.938	315,413
freemuxlet	A	Full	N/A	N/A	N/A	N/A
scSplit	A	Full	N/A	N/A	N/A	N/A
souporcell	B	Full	0.803	0.797	0.8	927,030
vireo	C	Full	0.866	0.847	0.866	267,370
CellBouncer		5000 cells	0.814	0.811	0.813	242,670
CellBouncer + refine_vcf		5000 cells	0.877	0.874	0.876	175,491
freemuxlet	B	5000 cells	0.769	0.769	0.769	148,899
scSplit	B,C	5000 cells	0.684	0.682	0.683	193,516
souporcell	B	5000 cells	0.792	0.782	0.787	193,869
vireo	C	5000 cells	0.866	0.85	0.858	68,411
CellBouncer		2000 cells	0.868	0.865	0.866	118,994
CellBouncer + refine_vcf		2000 cells	0.894	0.89	0.892	81,750
freemuxlet	B	2000 cells	0.756	0.756	0.756	64,583
scSplit	B,C	2000 cells	0.605	0.602	0.604	88,421
souporcell		2000 cells	0.916	0.903	0.909	67,832
vireo	C	2000 cells	0.851	0.837	0.844	23,870

Table S3. Accuracy of CellBouncer's MT-to-VCF pipeline compared to alternative clustering-based individual demultiplexing tools on a benchmarking dataset, related to Figure 3

Precision, recall, F score (harmonic mean of precision and recall), and number of informative variants produced by running several genotype-free individual demultiplexing tools on 10X Genomics 40,000 cell 7-donor NSCLC single-cell RNA-seq dataset[10]. Ground truth was the majority vote assignment across all methods plus 10X CellPlex data; ties were broken using CellPlex assignments. Number of variants is the number of output variants that differ among inferred individuals and could be used in downstream analyses. Error meanings: A: crashed (due to memory requirements) or never completed. B: clustering error: two inferred individuals correspond to one actual individual. C: many or all doublets are misassigned.

Source	Fraction	donor_1	donor_2	donor_3	donor_4	donor_5	donor_6	donor_7
donor_1	0.05	7.38E-02	4.05E-01	4.05E-01	4.05E-01	4.05E-01	4.05E-01	4.45E-01
donor_1	0.1	1.22E-04**	2.31E-01	5.01E-01	5.01E-01	2.31E-01	2.45E-01	5.36E-01
donor_1	0.15	1.04E-08**	6.93E-02	6.22E-01	6.22E-01	6.93E-02	6.93E-02	6.82E-01
donor_1	0.2	1.82E-13**	5.76E-02	9.80E-01	9.80E-01	8.06E-02	3.82E-02*	9.80E-01
donor_1	0.25	0.00E+00**	7.29E-03*	9.09E-01	9.09E-01	1.75E-02*	6.26E-03**	9.09E-01
donor_2	0.05	5.90E-01	5.90E-01	5.90E-01	5.90E-01	5.90E-01	5.90E-01	5.90E-01
donor_2	0.1	7.71E-01	2.90E-01	7.69E-01	7.71E-01	2.90E-01	4.95E-01	7.52E-01
donor_2	0.15	9.46E-01	1.38E-02*	9.01E-01	9.46E-01	8.29E-02	2.40E-01	9.01E-01
donor_2	0.2	9.79E-01	1.44E-04**	9.79E-01	9.79E-01	1.03E-02*	1.33E-01	9.79E-01
donor_2	0.25	8.56E-01	2.69E-07**	8.56E-01	8.56E-01	5.79E-04**	5.21E-02	8.56E-01
donor_3	0.05	3.82E-01	3.63E-01	4.85E-03**	3.63E-01	3.48E-01	3.48E-01	3.82E-01
donor_3	0.1	5.38E-01	1.29E-01	1.53E-08**	5.23E-01	7.54E-02	6.18E-02	5.38E-01
donor_3	0.15	7.37E-01	3.49E-02*	0.00E+00**	6.92E-01	8.98E-03**	2.51E-03**	7.37E-01
donor_3	0.2	8.68E-01	4.71E-03**	0.00E+00**	7.58E-01	5.72E-04**	1.79E-04**	8.68E-01
donor_3	0.25	9.32E-01	1.72E-03**	0.00E+00**	9.32E-01	1.16E-04**	9.71E-05**	9.32E-01
donor_4	0.05	4.41E-01	4.41E-01	4.41E-01	2.23E-02*	4.41E-01	4.41E-01	4.41E-01
donor_4	0.1	5.76E-01	2.83E-01	5.76E-01	5.42E-06**	1.96E-01	1.96E-01	5.76E-01
donor_4	0.15	8.32E-01	9.16E-02	7.95E-01	8.82E-12**	5.36E-02	3.17E-02*	8.32E-01
donor_4	0.2	9.46E-01	1.17E-02*	9.46E-01	0.00E+00**	5.62E-03**	3.00E-03**	9.46E-01
donor_4	0.25	8.25E-01	4.15E-03**	9.13E-01	0.00E+00**	2.77E-03**	2.77E-03**	8.77E-01
donor_5	0.05	6.70E-01	6.61E-01	6.61E-01	6.61E-01	6.61E-01	6.61E-01	6.61E-01
donor_5	0.1	9.23E-01	4.62E-01	9.23E-01	9.23E-01	1.69E-01	4.54E-01	9.23E-01
donor_5	0.15	9.04E-01	1.17E-01	9.04E-01	9.04E-01	9.17E-04**	1.17E-01	9.04E-01
donor_5	0.2	8.94E-01	7.88E-02	8.94E-01	8.94E-01	6.37E-07**	8.04E-03**	8.94E-01
donor_5	0.25	6.70E-01	2.55E-02*	6.93E-01	6.93E-01	2.01E-10*	8.71E-04**	6.70E-01
donor_6	0.05	6.17E-01	6.17E-01	6.17E-01	6.17E-01	6.17E-01	6.17E-01	6.17E-01
donor_6	0.1	7.60E-01	3.51E-01	7.60E-01	7.60E-01	2.50E-01	2.50E-01	7.60E-01
donor_6	0.15	9.10E-01	7.23E-02	8.49E-01	8.49E-01	3.03E-02*	6.26E-03**	9.10E-01
donor_6	0.2	9.23E-01	4.62E-02*	9.23E-01	9.23E-01	1.31E-02*	1.84E-04**	9.23E-01
donor_6	0.25	9.52E-01	1.29E-02*	9.52E-01	9.52E-01	2.35E-03**	1.07E-06**	9.52E-01
donor_7	0.05	4.19E-01	2.57E-01	3.24E-01	3.24E-01	2.57E-01	2.57E-01	4.51E-03**
donor_7	0.1	5.75E-01	5.64E-02	3.91E-01	4.35E-01	5.64E-02	3.92E-02*	1.07E-08**
donor_7	0.15	6.92E-01	6.42E-03**	3.99E-01	4.83E-01	7.60E-03**	2.69E-03**	9.33E-15**
donor_7	0.2	9.08E-01	2.01E-03**	7.95E-01	9.08E-01	3.09E-03**	8.10E-04**	0.00E+00**
donor_7	0.25	9.14E-01	4.74E-05**	6.88E-01	8.93E-01	7.27E-05**	8.26E-06**	0.00E+00**

Table S4. Tests for statistically significant deviations in inferred bulk pool proportions from baseline proportions in synthetic contamination experiment, related to Figure 4

Adjusted p-values for differences of bulk pool proportions from baseline proportions in synthetic contamination experiment. The first two columns denote the individual used as the source of introduced contamination and the percent of contaminant reads introduced to every cell. For each experiment, proportions of each individual were inferred using bulkprops and compared to the baseline proportions, inferred using bulkprops with 100 bootstrap replicates. P-values below a significance threshold of 0.05 are marked with asterisks; p-values below 0.01 are marked with two asterisks.

Mean reads per cell	Program	Mean	Min	Max
23	CellBouncer	0.8649	0.8647	0.8652
23	GMMdemux	0.8292	0.8285	0.8303
23	HTOdemux	0.8291	0.8289	0.8297
23	deMULTIplex2	0.7764	0.7715	0.7848
23	demuxmix	0.7128	0.7021	0.732
23	hashSolo	0.6807	0.6779	0.6824
23	hashedDrops	0.4881	0.4864	0.4913
57	CellBouncer	0.8871	0.8869	0.8874
57	GMMdemux	0.8839	0.8828	0.8851
57	HTOdemux	0.8739	0.8732	0.875
57	deMULTIplex2	0.8666	0.8659	0.8674
57	demuxmix	0.8618	0.8585	0.8671
57	hashSolo	0.8009	0.7869	0.8087
57	hashedDrops	0.7388	0.7378	0.7404
113	CellBouncer	0.893	0.8924	0.8942
113	GMMdemux	0.8927	0.8925	0.8928
113	HTOdemux	0.8896	0.8886	0.8911
113	deMULTIplex2	0.8892	0.8887	0.89
113	demuxmix	0.8804	0.8748	0.8836
113	hashSolo	0.8799	0.8781	0.8813
113	hashedDrops	0.8491	0.8484	0.8498
227	BFF	0.8959	0.8955	0.8966
227	CellBouncer	0.8945	0.8938	0.895
227	GMMdemux	0.8929	0.8924	0.8937
227	HTOdemux	0.8927	0.8918	0.8932
227	deMULTIplex2	0.8834	0.8826	0.8841
227	demuxmix	0.879	0.8786	0.8792
227	hashSolo	0.8784	0.864	0.8875
227	hashedDrops	0.8588	0.8575	0.8597
1126	BFF	0.8967	0.8966	0.8968
1126	CellBouncer	0.8936	0.8934	0.8941
1126	GMMdemux	0.8923	0.8918	0.8929
1126	HTOdemux	0.8843	0.8837	0.8847
1126	deMULTIplex2	0.875	0.8741	0.8757
1126	demuxmix	0.8737	0.8627	0.8949
1126	hashSolo	0.8716	0.8686	0.8753

1126	hashedDrops	0.8441	0.8431	0.8451
2235	BFF	0.8964	0.8962	0.8967
2235	CellBouncer	0.8932	0.8928	0.8937
2235	GMMdemux	0.8924	0.8921	0.8926
2235	HTOdemux	0.8808	0.8803	0.8813
2235	deMULTIplex2	0.875	0.8651	0.8938
2235	demuxmix	0.8722	0.872	0.8726
2235	hashSolo	0.8665	0.8568	0.8729
2235	hashedDrops	0.8007	0.8	0.8016
10504	BFF	0.896	0.896	0.8961
10504	CellBouncer	0.8927	0.8924	0.893
10504	GMMdemux	0.8927	0.8924	0.893
10504	HTOdemux	0.8767	0.8763	0.8769
10504	deMULTIplex2	0.8684	0.868	0.8687
10504	demuxmix	0.8675	0.8674	0.8676
10504	hashSolo	0.8519	0.8384	0.8599
10504	hashedDrops	0.6032	0.6028	0.6037
21296	BFF	0.8958	0.8958	0.8958
21296	CellBouncer	0.8927	0.8927	0.8927
21296	GMMdemux	0.8923	0.8923	0.8923
21296	HTOdemux	0.8759	0.8759	0.8759
21296	deMULTIplex2	0.8674	0.8674	0.8674
21296	demuxmix	0.8674	0.8674	0.8674
21296	hashSolo	0.8222	0.8222	0.8222
21296	hashedDrops	0.4869	0.4869	0.4869

Table S5. Accuracy of demux_tags compared to alternative programs on downsampled benchmarking dataset, related to Figure 5

F score (harmonic mean of precision and recall) of all HTO-assignment programs compared with CellBouncer demux_tags, on downsampled NSCLC datasets. Three trials were performed at each downsampling level, in which a subset of reads were randomly selected before counting HTO barcodes in reads. True labels came from the majority vote cell identity from running four different genotype-based demultiplexing programs on the same data, and discarding cells for which the majority identification was a tie. The mean, minimum, and maximum F score at each point are reported across the three trials. Results are sorted by decreasing F score within each level of downsampling.

Percent FG removed	Program	Mean	Min	Max
0.1	hashSolo	0.8941	0.8941	0.8941
0.1	BFF	0.8929	0.8929	0.8929
0.1	hashedDrops	0.8897	0.8897	0.8897
0.1	CellBouncer	0.8896	0.8896	0.8896
0.1	deMULTIplex2	0.8759	0.8759	0.8759
0.1	demuxmix	0.8691	0.8691	0.8691
0.1	HTOdemux	0.8071	0.8071	0.8071
0.1	GMMdemux	0.4868	0.4868	0.4868
0.25	BFF	0.8905	0.8905	0.8905
0.25	hashSolo	0.8904	0.8904	0.8904
0.25	hashedDrops	0.8863	0.8863	0.8863
0.25	CellBouncer	0.8835	0.8835	0.8835
0.25	deMULTIplex2	0.8723	0.8723	0.8723
0.25	demuxmix	0.8654	0.8654	0.8654
0.25	HTOdemux	0.7997	0.7997	0.7997
0.25	GMMdemux	0.4838	0.4838	0.4838
0.5	BFF	0.8899	0.8899	0.8899
0.5	hashSolo	0.8814	0.8814	0.8814
0.5	hashedDrops	0.8781	0.8781	0.8781
0.5	CellBouncer	0.8713	0.8713	0.8713
0.5	deMULTIplex2	0.8652	0.8652	0.8652
0.5	demuxmix	0.8548	0.8548	0.8548
0.5	HTOdemux	0.7815	0.7815	0.7815
0.5	GMMdemux	0.4745	0.4745	0.4745
0.75	hashedDrops	0.8461	0.8461	0.8461
0.75	hashSolo	0.8402	0.8402	0.8402
0.75	deMULTIplex2	0.8377	0.8377	0.8377
0.75	CellBouncer	0.8284	0.8284	0.8284
0.75	demuxmix	0.8153	0.8153	0.8153
0.75	BFF	0.7973	0.7973	0.7973
0.75	HTOdemux	0.7356	0.7356	0.7356
0.75	GMMdemux	0.4276	0.4275	0.4276
0.9	CellBouncer	0.7313	0.7313	0.7313
0.9	hashedDrops	0.6871	0.6871	0.6871
0.9	hashSolo	0.6831	0.6831	0.6831
0.9	deMULTIplex2	0.6673	0.6673	0.6673

0.9	HTOdemux	0.548	0.548	0.548
0.9	demuxmix	0.4981	0.4077	0.6588
0.9	BFF	0.4106	0.4106	0.4106
0.9	GMMdemux	0.3256	0.3227	0.3272

Table S6. Accuracy of demux_tags compared to alternative programs on decreased signal-to-noise benchmarking dataset, related to Figure 5

F score (harmonic mean of precision and recall) of all HTO-assignment programs compared with CellBouncer demux_tags, on decreased signal-to-noise NSCLC datasets. Three trials were performed at each level, in which a percent of reads from the tag(s) representing the true cell identity were removed (first column). True labels came from the majority vote cell identity from running four different genotype-based demultiplexing programs on the same data, and discarding cells for which the majority identification was a tie. The mean, minimum, and maximum F score at each point are reported across the three trials. Results are sorted by decreasing F score within each level.

library	composite_line	human_contrib1	human_contrib2	chimp_contrib1	chimp_contrib2	bonob_contrib
A1_A2	2	H20961	H28126			
A1_A2	4	H20961		C8861		
A1_A2	29			C40670	C40670	
A1_A2	34			C3624	C8861	
A1_A2	61	H20961		C40670		
A1_A2	80	H21792		C40280		
A1_A2	83			C40670	C6007B	
A1_A2	88	H21792		C40280		
A1_A2	91	H21792	H29089			
A1_A2	96	H29089		C3651		
A1_A2	106			C40280	C40280	
A1_A2	127	H20961		C3651		
A1_A2	131			C40670		CongoA4B
A1_A2	151			C8861		CongoA4B
B	1	H20961	H20961			
B	17	H23555		C40670		
B	27	H23555		C40670		
B	33	H23555		C3624		
B	35			C3624	C40670	
B	39	H20961	H23555			
B	58			C8861	C8861	
B	73	H20961		C6007B		
B	123	H21792	H23555			
B	154			C40670		CongoA4B

Table S7. Composite cell lines used in this study, related to STAR Methods

Composite cell lines created for this study. Each line was created through polyethylene glycol fusion of two diploid contributor cell lines. This study included five human/human autotetraploid lines (including one self-self fusion, in which contributor lines were identical), six chimpanzee/chimpanzee autotetraploid lines including three self-self fusions, ten allotetraploid human/chimpanzee lines encompassing nine distinct genotypic combinations, and three allotetraploid chimpanzee/bonobo lines encompassing two distinct genotypic combinations.

Library	Program	Precision	Recall	F
A1	CellBouncer	0.96	0.95	0.955
A1	demuxalot	0.945	0.945	0.945
A1	demuxlet	N/A	N/A	N/A
A1	Vireo	0.26	0.214	0.235
A2	CellBouncer	0.857	0.841	0.849
A2	demuxalot	0.827	0.827	0.827
A2	demuxlet	N/A	N/A	N/A
A2	Vireo	0.24	0.19	0.212
B	CellBouncer	0.592	0.589	0.591
B	demuxalot	0.577	0.577	0.577
B	demuxlet	N/A	N/A	N/A
B	Vireo	0.116	0.0874	0.1

Table S8. Agreement of MULTIseq-inferred cell identities with genotype-based demultiplexing calls on composite iPSC data, related to STAR Methods

Agreement of MULTIseq labels tagging cell line identity (used as truth) with results of genotype-based demultiplexing of cells from inter- and intra-species tetraploid composite cell lines. Because of genotype demultiplexing tools' inability to identify droplets containing more than two genomes, cells inferred to be doublets from MULTIseq data were excluded. A1 and A2 are technical replicates of the same library. N/A values indicate that the program did not run in the allowed time frame (12 days).

References

1. Edenhofer, F.C., Térmeg, A., Ohnuki, M., Jocher, J., Kliesmete, Z., Briem, E., Hellmann, I., and Enard, W. (2024). Generation and characterization of inducible KRAB-dCas9 iPSCs from primates for cross-species CRISPRi. iScience *27*, 110090. https://doi.org/10.1016/j.isci.2024.110090.

2. Hartoularos, G.C., Si, Y., Zhang, F., Kathail, P., Lee, D.S., Ogorodnikov, A., Sun, Y., Song, Y.S., Kang, H.M., and Ye, C.J. (2023). Reference-free multiplexed single-cell sequencing identifies genetic modifiers of the human immune response. Preprint at bioRxiv, https://doi.org/10.1101/2023.05.29.542756.

3. Xu, J., Falconer, C., Nguyen, Q., Crawford, J., McKinnon, B.D., Mortlock, S., Senabouth, A., Andersen, S., Chiu, H.S., Jiang, L., et al. (2019). Genotype-free demultiplexing of pooled single-cell RNA-seq. Genome Biol. *20*, 290. https://doi.org/10.1186/s13059-019-1852-7.

4. Heaton, H., Talman, A.M., Knights, A., Imaz, M., Gaffney, D.J., Durbin, R., Hemberg, M., and Lawniczak, M.K.N. (2020). Souporcell: robust clustering of single-cell RNA-seq data by genotype without reference genotypes. Nat. Methods *17*, 615–620. https://doi.org/10.1038/s41592-020-0820-1.

5. Huang, Y., McCarthy, D.J., and Stegle, O. (2019). Vireo: Bayesian demultiplexing of pooled single-cell RNA-seq data without genotype reference. Genome Biol. *20*, 273. https://doi.org/10.1186/s13059-019-1865-2.

6. Lex, A., Gehlenborg, N., Strobelt, H., Vuillemot, R., and Pfister, H. (2014). UpSet: Visualization of intersecting sets. IEEE Trans. Vis. Comput. Graph. *20*, 1983–1992. https://doi.org/10.1109/TVCG.2014.2346248.

7. Szklarczyk, D., Kirsch, R., Koutrouli, M., Nastou, K., Mehryary, F., Hachilif, R., Gable, A.L., Fang, T., Doncheva, N.T., Pyysalo, S., et al. (2023). The STRING database in 2023: protein-protein association networks and functional enrichment analyses for any sequenced genome of interest. Nucleic Acids Res. *51*, D638–D646. https://doi.org/10.1093/nar/gkac1000.

8. Lorenz, R., Bernhart, S.H., Höner Zu Siederdissen, C., Tafer, H., Flamm, C., Stadler, P.F., and Hofacker, I.L. (2011). ViennaRNA Package 2.0. Algorithms Mol. Biol. *6*, 26. https://doi.org/10.1186/1748-7188-6-26.

9. Kamath, T., Abdulraouf, A., Burris, S.J., Langlieb, J., Gazestani, V., Nadaf, N.M., Balderrama, K., Vanderburg, C., and Macosko, E.Z. (2022). Single-cell genomic profiling of human dopamine neurons identifies a population that selectively degenerates in Parkinson's disease. Nat. Neurosci. *25*, 588–595. https://doi.org/10.1038/s41593-022-01061-1.

10. 10X Genomics (2021). 40k Mixture of NSCLC DTCs from 7 donors, 3' HT v3.1. https://www.10xgenomics.com/datasets/40-k-mixture-of-nsclc-dt-cs-from-7-donors-3-ht-v-3-1-3-1-high-6-1-0.
